# A particle swarm based hybrid system for imbalanced medical data sampling

**DOI:** 10.1186/1471-2164-10-S3-S34

**Published:** 2009-12-03

**Authors:** Pengyi Yang, Liang Xu, Bing B Zhou, Zili Zhang, Albert Y Zomaya

**Affiliations:** 1 School of Information Technologies (J12), The University of Sydney, NSW 2006, Australia; 2 National ICT Australia, Australian Technology Park, Eveleigh, NSW 2015, Australia; 3 Faculty of Computer and Information Science, Southwest University, CQ 400715, PR China; 4 School of Information Technology, Deakin University, Geelong, VIC 3217, Australia; 5 Sydney Bioinformatics Center and the Center for Mathematical Biology, The University of Sydney, NSW 2006, Australia

## Abstract

**Background:**

Medical and biological data are commonly with small sample size, missing values, and most importantly, imbalanced class distribution. In this study we propose a particle swarm based hybrid system for remedying the class imbalance problem in medical and biological data mining. This hybrid system combines the particle swarm optimization (PSO) algorithm with multiple classifiers and evaluation metrics for evaluation fusion. Samples from the majority class are ranked using multiple objectives according to their merit in compensating the class imbalance, and then combined with the minority class to form a balanced dataset.

**Results:**

One important finding of this study is that different classifiers and metrics often provide different evaluation results. Nevertheless, the proposed hybrid system demonstrates consistent improvements over several alternative methods with three different metrics. The sampling results also demonstrate good generalization on different types of classification algorithms, indicating the advantage of information fusion applied in the hybrid system.

**Conclusion:**

The experimental results demonstrate that unlike many currently available methods which often perform unevenly with different datasets the proposed hybrid system has a better generalization property which alleviates the method-data dependency problem. From the biological perspective, the system provides indication for further investigation of the highly ranked samples, which may result in the discovery of new conditions or disease subtypes.

## Background

One of the difficulties in medical and biological data analysis is the highly skewed class distribution of different sample types. This could happen when special cases or "positive" samples are of limited size, while control or "negative" samples are more abundant [1-4]. Sometimes, disease samples are divided into subtypes, with some of which are common while others are very rare. Samples from those rare subtypes are represented as minority classes which also cause the imbalance of the class distribution [5]. Here the challenge is how to precisely and correctly classify the minority samples (rare cases) because they often carry important biological implications but tend to be ignored by the classification model which is overwhelmed by the majority samples. In data mining community this problem is known as imbalanced data classification [6] and recently received an increasing attention for its practical importance.

There are mainly two strategies in dealing with imbalanced data learning: via sampling and via cost-sensitive learning. Although cost-sensitive learning does not modify the data distribution or introduce duplicated samples, it requires the right cost-metric to assign different penalties for misclassification of different sample types. However, the correct cost-metric is often unknown a priori for a given dataset, and an improper cost-metric can significantly degenerate the classification accuracy [7]. Recently, much effort has been made for developing new sampling strategies [8-10].

Data sampling strategies can often be categorized into two groups: oversampling and undersampling. In oversampling, the samples in the minority class are increased to match the samples of the majority class, while in undersampling the samples in the majority class are decreased to match the samples of the minority class. The classical or "naive" method is to randomly select samples from minority class and use the selected samples to increase the size of the minority class for oversampling (random oversampling) or to randomly select samples from majority class and remove them so as to decrease the sample size for undersampling (random undersampling) [11]. More advanced methods attempt to employ certain intelligent strategies such as clustering [10], working on the decision boundary [12] or synthesizing new examples based on the data characteristics [8,13]. There are also many distance-based methods which try to select the samples with the nearest distance or farthest distance between the majority class and the minority class [14]. However, currently there is no clear way to determine which rule should be followed, and simply applying random sampling often beats those "smart" methods [15]. The unsuccessful experiences imply that those methods are largely data-depended. Therefore, designing more flexible and better generalized algorithms which are self-adaptable to different data patterns in imbalanced data sampling and accurate model construction is clearly a desirable goal. This is particularly true in medical data classification and diagnosis because a false positive prediction will cause unwarranted worries while a false negative prediction will increase the risk of missing medical attention.

In previous work, Zhang and Yang successfully applied a genetic ensemble hybrid system to the feature selection of high-dimensional data [16]. If we convert the question by treating samples as features and re-adopt such kind of feature selection methods to select a subset of samples in majority class for building a balanced classification model, will such formulation lead to a better balanced classification result? This study is set out to investigate this quest. Here we formulate the problem as an optimization process and employ the particle swarm optimization (PSO) algorithm as the sample selection strategy [17,18]. Multiple classification algorithms with several most indicative metrics for imbalance classification measurement are used as multiple objectives to guide the sample selection process. Although there are continuing debates on which technique is better [19], undersampling is often preferred because no duplicated samples are introduced [20,21]. Therefore, our study will concentrate on selecting an optimal subset of majority samples and combine them with the minority samples for building a balanced classification model. Nevertheless, the proposed algorithm can be easily applied to oversampling by changing the target as minority samples.

## Methods

### System overview

**Figure 1:**
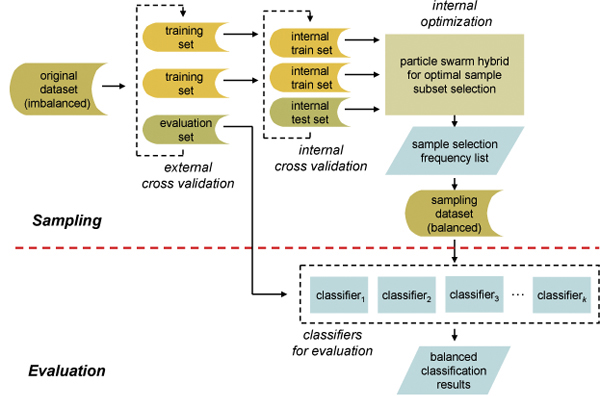
**Schematic flow chart of sampling and evaluation processes**. The original imbalanced dataset are split to training and test sets with an external stratified cross validation. The sampling process is then conducted on an internal stratified cross validation for creating a balanced training set. The classification models are built on the balanced training set and the test set from the external cross validation is classified using the obtained classification models.

The problem of using highly imbalanced dataset for pattern recognition is that the classification model built on the training data tends to be biased on preferring the majority class while ignoring the samples from the minority class. Data sampling method tries to remedy the skewed class distribution by either increasing the sample size of minority class or decreasing the sample size of majority class. However, algorithms that modify the sample distribution with greedy measures can introduce undesired bias. In this study we re-apply the techniques in feature selection to data sampling using a PSO based hybrid system. The schematic flow of sampling and evaluation processes in our hybrid system is illustrated in Figure 1.

As can be seen, the work flow can be divided into two steps, namely, sampling and evaluation. For a given dataset, an external 3-fold stratified cross validation is applied to partition the dataset into external training sets (sampling sets) and external test sets (evaluation sets). Then, the external training sets are further partitioned with an internal 3-fold stratified cross validation, which gives the internal training sets and internal test sets. The internal training sets are used for sampling, while the internal test sets are used for guiding the optimization process. The external test sets are reserved for evaluation of the balanced dataset and is excluded from the sampling procedure.

In the sampling procedure, the PSO hybrid system is used to evaluate the merit of each sample from the majority class in compensating the class imbalance. This is accomplished by generating different sample subsets of majority class and combining them with samples from the minority class for classification model construction and then for internal test fold classification. Those subsets that can create more accurate classification models are favored and optimized in each PSO iteration. When the termination criterion is met, selected samples from the last iteration are ranked by their selection frequency. After the sample selection frequency list is obtained, a balanced dataset can be created by combining the highly ranked samples of majority class with samples of minority class. In the evaluation step, different classification models are created using the balanced dataset generated by PSO hybrid system, and the external evaluation dataset is applied to evaluate the classification accuracy with different evaluation metrics. Such a training and evaluation process keeps the evaluation dataset for independent validation, which provides an unbiased evaluation.

### Particle swarm based optimization

Particle swarm optimization (PSO) is a new group of population-based algorithms which uses the idea of social communication and historical behaviors to adjust the optimization process [17]. It possesses the advantages such as high-performance and global optimization, which make it very popular in many biological related applications. Specifically, Lee combined PSO with Genetic Algorithm (GA) and Support Vector Machine (SVM) for gene selection of microarray data [22], Xu et al. used PSO to optimize the structure of Recurrent Neural Network (RNN) in gene network modeling [23], while Rasmussen and Krink applied PSO for Hidden Markov Model optimization in multiple sequence alignment [24]. In our system, a binary version of PSO (BPSO) [18,25] is employed for a new application, in which BPSO is hybridized with multiple classifiers and metrics for data sample selection and ranking.

**Figure 2:**
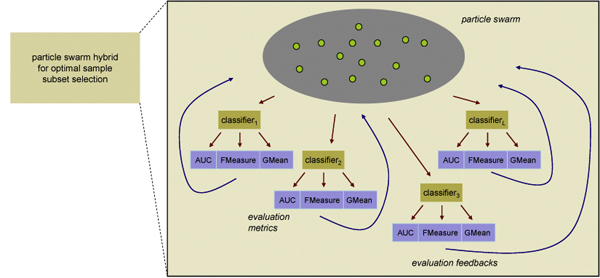
**Particle swarm based hybrid module for data sampling**. Multiple classification algorithms are used to guide the sampling process. Within each classification algorithm, three evaluation metrics are employed to evaluated the goodness of the sample subsets. PSO algorithm is used to optimize the sample subsets according to the evaluation results of each classification component.

Figure 2 gives a graphical representation of this particle swarm based hybrid module. In this module, different sample subsets are encoded as particles, and each particle is evaluated by multiple classifiers each with three evaluation metrics. The system seeks for the sample subsets that present good classification accuracy with not only a certain type of classifier but a wide range of them each provides the feedback using several evaluation criteria. The use of this hybrid system is justified with the argument that multiple criteria formulation is preferable than a single classification algorithm or evaluation metric because the results produced in this way will have a better generalization property.

Each sample of majority class in the training dataset is assigned an index in the particle space. The locus equals "1" if the sample is selected for building classification model or equals "0" if the sample is excluded from building the classification model. Suppose we have a population of *n *particles, with *i *be the index of a particle in the swarm (*i *= 1, ..., *n*), *j *be the index of dimension in the particle (*j *= 1, ..., *m*), and *t *be the counter of iterations. The velocity of the *i*th particle *v*_*i*, *j*_(*t*) and the position of this particle *x*_*i*, *j*_(*t*) is updated by BPSO with following equations:

where *pbest*_*i*, *j *_and *gbest*_*i*, *j *_are the previous best position and the best position found by informants, respectively. *random*() is the pseudo-random number generator that creates uniform distribution between [0-1].

### Fitness and evaluation metrics

Fitness function is the optimization guide of the BPSO. It governs the update of *pbest*_*i*, *j *_and *gbest*_*i*, *j*_. It has been pointed out that in the imbalanced data evaluation a simple classification accuracy is not an indicative measure because the accuracy value is profoundly influenced by the large class [13].

Alternatively, metrics including Area Under the ROC Curve (AUC), F-Measure (FMeasure), and Geometric Mean (GMean) are often chosen as more appropriate measures [10,12,26,27]. Here we combine multiple evaluation metrics in BPSO fitness function, which is defined as follows:

where *L *is the number of classifiers integrated in the hybrid system and *fitness*_*i*_(*s*) is formulated as follows:

where *s *is the sample subset to be evaluated. This fitness function is essentially a weighted combination of the above three evaluation metrics, *AUC*(*s*) is calculated using Mann Whitney statistic [28], while *FMeasure*(*s*) and *GMean*(*s*) are calculated as follows:

where each component in *FMeasure*(*s*) and *GMean*(*s*) is further defined as follows:

Precision:

Sensitivity or Recall:

Specificity:

where *N*_*TP *_is the number of true positive, *N*_*TN *_is the number of true negative, *N*_*FN *_is the number of false negative, and *N*_*FP *_is the number of false positive.

### Classifiers

One limitation of previous efforts on imbalanced data analysis is that most studies only focused on Decision Tree as evaluation criterion [6]. Instead of choosing certain type of classification algorithm for evaluation, multiple classifiers have been incorporated in our particle swarm based hybrid system. The reason of utilizing multiple classifiers is to balance multiple classification hypotheses so as to reveal true improvement of the sampling dataset.

Specifically, the classification algorithms employed in the hybrid system composition includes Decision Tree (J48), *k*-Nearest Neighbor (*k*NN), Naive Bayes (NB), Random Forest (RF) and Logistic Regression (LOG). J48 is a widely used decision tree classifier. It approximates discrete-valued functions and a group of favorite features selected by the algorithm are used as the test points at the tree nodes. Each path of the node is then created for partitioning the value of the feature. *k*NN classifier calculates the similarity, which is called distance, of a given instance with the others and assign the given instance into the majority class which the *k *most similar instances belong to. Such similarity can be defined as Euclidean distance, Manhattan distance or Pearson correlation. Naive bayes classifier bases its learning strategy on probability theory. It tries to estimate the distribution of the data and classify a sample by assigning the sample into a class with the highest probability. Random forest, as its name indicates, is a collection of decision trees [29]. Instead of using a single tree to make the classification, Random forest algorithm combines the decisions of several trees each trained on a feature subset of the original dataset. Lastly, the Logistic Regression classifier uses a logistic function to compute the coefficients of input features with respect to the class label. It has been used extensively in modeling binomially distributed data.

### Main loop

**Figure 3:**
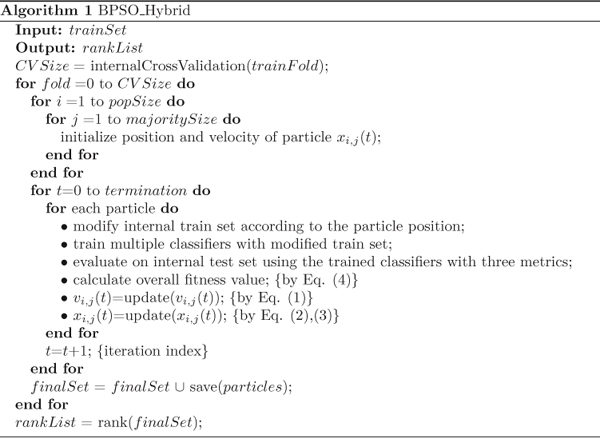
**The main loop of the BPSO based hybrid algorithm**.

Putting above components together, the BPSO based hybrid system can be summarized by pseudo-code in Figure 3.

## Experimental settings

### Datasets

Four typical medical datasets are obtained from UCI Machine Learning Repository [30] and a genome wide association study (GWAS) dataset is obtained from the genotyping of Single Nucleotide Polymorphisms (SNPs) of Age-related Macular Degeneration (AMD) [31].

For the medical data, the first dataset named "Blood" was generated by Blood Transfusion Service Center in Taiwan. It has 568 samples denoted as not donating blood and 180 as donating blood in March 2007, and the prevalence of the dataset is 24.1%. The task is to classify these samples based on the information of blood donation frequency, recency etc. The second dataset, "Survival", was generated from the survey conducted on the survival of patients who had undergone surgery for breast cancer. It contains 225 patients who survived 5 years or longer and 81 patients died within 5 year. The prevalence of this dataset is 26.5%. The third dataset with the name of "Diabetes" is obtained from the study of diabetes in Pima Indian population. 500 samples were identified as negative while the other 268 samples were identified as positive, which gives the prevalence of 34.9%. The last dataset called "Breast" was created for breast tumor diagnosis. Within this dataset, 151 are benign samples and 47 are malignant samples, and the prevalence is 23.7%.

The GWAS dataset contains 146 samples with each sample been described by more than 100,000 SNPs. Within the 146 samples 46 are labeled as geographic atrophy either central or non-central to the macula (CGA), 50 are labeled as uniocular choroidal neovascularization (Neov), and the rest 50 are the control samples. Therefore, the task is divided to classify CGA samples from the rest (which gives a prevalence of minority class of 31.5%) and to classify Neov samples from the rest (which gives a prevalence of minority class of 34.2%). In SNPs selection, we applied the selection procedure utilized by Chen et al. [32], and obtained 17 SNPs from two Linkage Disequilibrium (LD) blocks. They are rs2019727, rs10489456, rs3753396, rs380390, rs2284664, and rs1329428 from the first block, and rs4723261, rs764127, rs10486519, rs964707, rs10254116, rs10486521, rs10272438, rs10486523, 10486524, rs10486525, and rs1420150 from the second block. Based on previous investigation of AMD [33-35], we added another six SNPs to avoid analysis bias. They are rs800292, rs1061170, rs1065489, rs1049024, rs2736911, and rs10490924. Moreover, environment factors of Smoking status and Sex are also encoded into each dataset due to their high association to the AMD development. Together, we formed the two subtype datasets with each sample represented as 25 factors.

The summary of each dataset is given in Table 1.

**Table 1:** Summary of the medical and biological datasets used in the experiments.

Dataset	# Feature	# Negative	# Positive	Prevalence
Blood	4	568	180	24.1%
Survival	3	225	81	26.5%
Diabetes	8	500	268	34.9%
Breast	32	151	47	23.7%
AMD-CGA	25	100	46	31.5%
AMD-Neov	25	96	50	34.2%

### Implementation

We compare our particle swarm based sampling strategy with random undersampling, random oversampling, and clustering based sampling. Random undersampling and random oversampling are implemented by decreasing samples of majority class or increasing samples of minority class to match the counterpart with a uniformed possibility, respectively. Clustering based sampling is implemented as the base version of those described in [10], that is, to cluster the data samples with *k*-mean algorithm and randomly select samples of majority class according to the majority/minority ratio of each cluster and the cluster size. We used the *k *size of 10 for *k*-mean clustering and the Euclidean distance for similarity calculation.

As per the particle swarm based hybrid system, we code the particle space as an *m *dimension space with *m *equals to the size of the majority samples in the training set. Different parameter settings of the particle swarm component are investigated empirically, and we fix the best combination (as shown in Table 2) for evaluation and comparison. Different classification algorithms are implemented by using APIs of the WEKA machine learning suite [28] through the main code.

**Table 2:** Parameter settings of the particle swarm based hybrid system.

Parameter	Value
Size of Classification Committee	5
Number of Evaluation Metrics	3
Size of Particle Population	100
Iteration	150
Update Rule	Sigmoid Function
Cognitive Constant	1.43
Social Acceleration Constant	1.43
Inertia Weight	0.689
Velocity Bound	0.018-0.982
Fitness Weight	*w*_1 _= *w*_2 _= *w*_3 _= 1/3

## Results

Tables 3, 4, 5, 6, 7, 8 provide the evaluation details of each sampling method on each dataset, respectively. All results are obtained by averaging three independent trials on each dataset. We named particle swarm based hybrid system as "PSO", random undersampling as "RU", random oversampling as "RO", and clustering based sampling as "Cluster" for convenience. For each sampling method, the evaluation results are presented with respect to 3 evaluation metrics and 10 different classification algorithms including Decision Tree (J48), 3-Nearest Neighbor (3NN), Naive Bayes (NB), Random Forest with 5 trees (RF5), Logistic Regression (LOG), 1-Nearest Neighbor (1NN), 7-Nearest Neighbor (7NN), Sequential Minimal Optimization of Support Vector Machine (SMO), Random Forest with 10 trees (RF10), and Radial Basis Function Network (RBFNet). With a careful observation it is clear that in most cases PSO achieved better classification accuracy using all three evaluation metrics in comparison with the other three sampling methods. This can be further confirmed by averaging across different classification results with respect to each evaluation metric (indicated in column "R. Avg." of Tables 3, 4, 5, 6, 7, 8). Also observed is that the improvement is essentially consistent across 10 different types of classifiers. This can be seen from the row "C. Avg." of Tables 3, 4, 5, 6, 7, 8. It should be noted that only the first five classifiers are used in PSO optimization and data sampling, while the last five classifiers are only used for evaluating the generation property of the hybrid system. Also, the evaluation is done on the independent test set through external cross validation. Therefore, it is safe to draw a conclusion that re-sampling dataset using PSO can lead to a higher data sampling quality and better generalization property. For random undersampling and random oversampling we found that random undersampling is more effective, albeit in a few cases random oversampling appear to be quite competitive. As to clustering based sampling, it performs competitively to random under- and oversampling in "Diabetes", "Breast", "AMD-CGA", and "AMD-Neov" datasets but relatively poor on "Blood" and "Survival" datasets.

**Table 3:** Evaluation results of Blood dataset using different sampling strategies with three metrics across ten classification algorithms.

Method	Metric	Classifier										
		
		J48	3NN	NB	RF5	LOG	1NN	7NN	SMO	RF10	RBFNet	R. Avg.
PSO	AUC	0.693	0.656	0.706	0.656	0.736	0.612	0.696	0.667	0.660	0.720	0.680
	FMeasure	0.495	0.446	0.458	0.430	0.494	0.409	0.485	0.486	0.434	0.487	0.462
	GMean	0.671	0.622	0.634	0.605	0.668	0.590	0.662	0.655	0.614	0.663	0.638
	
	C. Avg.	0.620	0.575	0.599	0.564	0.633	0.537	0.614	0.603	0.569	0.623	0.593

RU	AUC	0.663	0.647	0.713	0.632	0.745	0.597	0.689	0.666	0.638	0.710	0.669
	FMeasure	0.474	0.425	0.417	0.419	0.511	0.393	0.461	0.486	0.424	0.462	0.447
	GMean	0.643	0.609	0.586	0.600	0.686	0.577	0.641	0.655	0.605	0.639	0.624
	
	C. Avg.	0.593	0.560	0.572	0.550	0.647	0.522	0.597	0.602	0.556	0.604	0.580

RO	AUC	0.657	0.635	0.710	0.618	0.749	0.573	0.652	0.671	0.629	0.715	0.661
	FMeasure	0.460	0.422	0.375	0.387	0.514	0.339	0.432	0.491	0.380	0.474	0.428
	GMean	0.635	0.607	0.538	0.568	0.689	0.522	0.615	0.663	0.561	0.651	0.605
	
	C. Avg.	0.584	0.555	0.541	0.524	0.651	0.478	0.566	0.608	0.523	0.613	0.565

Cluster	AUC	0.616	0.660	0.677	0.614	0.651	0.571	0.661	0.658	0.629	0.711	0.645
	FMeasure	0.449	0.420	0.382	0.405	0.429	0.318	0.414	0.419	0.348	0.454	0.404
	GMean	0.587	0.583	0.556	0.559	0.560	0.534	0.616	0.635	0.608	0.658	0.590
	
	C. Avg.	0.551	0.554	0.538	0.526	0.547	0.474	0.564	0.571	0.528	0.608	0.546

**Table 4:** Evaluation results of Survival dataset using different sampling strategies with three metrics across ten classification algorithms.

Method	Metric	Classifier										
		
		J48	3NN	NB	RF5	LOG	1NN	7NN	SMO	RF10	RBFNet	R. Avg.
PSO	AUC	0.660	0.626	0.660	0.668	0.698	0.614	0.618	0.617	0.680	0.700	0.654
	FMeasure	0.495	0.422	0.495	0.469	0.542	0.459	0.421	0.447	0.464	0.492	0.471
	GMean	0.641	0.580	0.635	0.618	0.687	0.612	0.581	0.587	0.614	0.643	0.620
	
	C. Avg.	0.599	0.543	0.597	0.585	0.642	0.562	0.540	0.550	0.586	0.612	0.582

RU	AUC	0.636	0.565	0.633	0.628	0.668	0.589	0.619	0.586	0.659	0.651	0.623
	FMeasure	0.482	0.406	0.459	0.455	0.486	0.436	0.429	0.399	0.460	0.477	0.449
	GMean	0.626	0.562	0.598	0.599	0.637	0.589	0.587	0.554	0.608	0.619	0.598
	
	C. Avg.	0.581	0.511	0.563	0.561	0.597	0.538	0.545	0.513	0.576	0.582	0.557

RO	AUC	0.619	0.617	0.641	0.631	0.684	0.588	0.602	0.615	0.639	0.663	0.631
	FMeasure	0.465	0.433	0.427	0.389	0.487	0.354	0.413	0.411	0.368	0.459	0.422
	GMean	0.617	0.579	0.561	0.553	0.632	0.514	0.573	0.547	0.534	0.608	0.574
	
	C. Avg.	0.567	0.543	0.543	0.524	0.601	0.485	0.529	0.524	0.514	0.577	0.542

Cluster	AUC	0.623	0.564	0.642	0.601	0.664	0.546	0.570	0.595	0.616	0.634	0.606
	FMeasure	0.451	0.376	0.443	0.366	0.460	0.325	0.397	0.380	0.389	0.436	0.402
	GMean	0.602	0.538	0.559	0.539	0.636	0.497	0.546	0.512	0.570	0.601	0.560
	
	C. Avg.	0.559	0.493	0.548	0.502	0.587	0.456	0.504	0.496	0.525	0.557	0.523

**Table 5:** Evaluation results of Diabetes dataset using different sampling strategies with three metrics across ten classification algorithms.

Method	Metric	Classifier										
		
		J48	3NN	NB	RF5	LOG	1NN	7NN	SMO	RF10	RBFNet	R. Avg.
PSO	AUC	0.746	0.761	0.808	0.801	0.827	0.693	0.793	0.740	0.817	0.786	0.777
	FMeasure	0.660	0.618	0.638	0.662	0.661	0.612	0.651	0.662	0.671	0.639	0.647
	GMean	0.734	0.698	0.717	0.736	0.734	0.691	0.727	0.738	0.745	0.719	0.724
	
	C. Avg.	0.713	0.692	0.721	0.733	0.741	0.665	0.724	0.713	0.744	0.715	0.716

RU	AUC	0.697	0.739	0.801	0.765	0.829	0.665	0.773	0.737	0.791	0.759	0.756
	FMeasure	0.635	0.603	0.635	0.628	0.665	0.581	0.636	0.657	0.659	0.609	0.631
	GMean	0.707	0.686	0.714	0.705	0.740	0.660	0.714	0.734	0.734	0.694	0.709
	
	C. Avg.	0.680	0.676	0.717	0.699	0.745	0.635	0.708	0.709	0.728	0.687	0.699

RO	AUC	0.709	0.722	0.799	0.774	0.831	0.653	0.774	0.735	0.796	0.797	0.760
	FMeasure	0.634	0.592	0.628	0.612	0.665	0.549	0.621	0.656	0.616	0.636	0.622
	GMean	0.713	0.675	0.705	0.696	0.739	0.643	0.699	0.733	0.696	0.716	0.702
	
	C. Avg.	0.685	0.663	0.711	0.694	0.745	0.615	0.698	0.708	0.703	0.716	0.695

Cluster	AUC	0.701	0.729	0.801	0.759	0.813	0.608	0.769	0.753	0.788	0.784	0.751
	FMeasure	0.624	0.603	0.635	0.598	0.684	0.513	0.626	0.680	0.643	0.629	0.624
	GMean	0.702	0.678	0.711	0.686	0.723	0.615	0.698	0.752	0.721	0.711	0.700
	
	C. Avg.	0.676	0.670	0.716	0.681	0.740	0.579	0.698	0.728	0.717	0.708	0.692

**Table 6:** Evaluation results of Breast dataset using different sampling strategies with three metrics across ten classification algorithms.

Method	Metric	Classifier										
		
		J48	3NN	NB	RF5	LOG	1NN	7NN	SMO	RF10	RBFNet	R. Avg.
PSO	AUC	0.580	0.610	0.602	0.603	0.736	0.570	0.625	0.670	0.637	0.549	0.618
	FMeasure	0.418	0.423	0.393	0.369	0.489	0.394	0.425	0.487	0.392	0.359	0.415
	GMean	0.593	0.599	0.577	0.550	0.661	0.553	0.595	0.660	0.577	0.544	0.591
	
	C. Avg.	0.530	0.544	0.524	0.507	0.629	0.506	0.548	0.606	0.535	0.484	0.541

RU	AUC	0.562	0.597	0.587	0.604	0.722	0.515	0.612	0.650	0.639	0.568	0.606
	FMeasure	0.393	0.407	0.378	0.403	0.479	0.345	0.422	0.466	0.399	0.388	0.408
	GMean	0.552	0.583	0.558	0.571	0.656	0.502	0.597	0.637	0.581	0.568	0.581
	
	C. Avg.	0.502	0.529	0.508	0.526	0.619	0.454	0.544	0.584	0.540	0.508	0.532

RO	AUC	0.569	0.564	0.596	0.593	0.789	0.544	0.582	0.688	0.639	0.480	0.604
	FMeasure	0.327	0.391	0.384	0.354	0.560	0.325	0.382	0.509	0.297	0.286	0.382
	GMean	0.508	0.565	0.569	0.522	0.701	0.512	0.560	0.683	0.452	0.475	0.555
	
	C. Avg.	0.468	0.507	0.516	0.490	0.683	0.460	0.508	0.627	0.463	0.414	0.514

Cluster	AUC	0.543	0.616	0.538	0.554	0.711	0.547	0.603	0.641	0.586	0.528	0.587
	FMeasure	0.384	0.402	0.356	0.325	0.466	0.371	0.417	0.450	0.342	0.332	0.385
	GMean	0.579	0.585	0.550	0.508	0.648	0.527	0.583	0.638	0.540	0.529	0.569
	
	C. Avg.	0.502	0.534	0.481	0.462	0.608	0.482	0.534	0.576	0.489	0.463	0.514

**Table 7:** Evaluation results of AMD-CGA dataset using different sampling strategies with three metrics across ten classification algorithms.

Method	Metric	Classifier										
		
		J48	3NN	NB	RF5	LOG	1NN	7NN	SMO	RF10	RBFNet	R. Avg.
PSO	AUC	0.609	0.559	0.606	0.591	0.566	0.599	0.590	0.572	0.573	0.540	0.581
	FMeasure	0.481	0.462	0.489	0.464	0.448	0.485	0.468	0.460	0.465	0.455	0.468
	GMean	0.580	0.557	0.572	0.551	0.538	0.576	0.573	0.550	0.545	0.539	0.558
	
	C. Avg.	0.557	0.526	0.556	0.535	0.517	0.553	0.544	0.527	0.528	0.511	0.536

RU	AUC	0.569	0.547	0.549	0.594	0.567	0.569	0.579	0.556	0.604	0.570	0.570
	FMeasure	0.434	0.457	0.439	0.476	0.453	0.439	0.446	0.417	0.456	0.446	0.446
	GMean	0.538	0.556	0.538	0.580	0.551	0.547	0.553	0.523	0.550	0.549	0.549
	
	C. Avg.	0.514	0.520	0.509	0.550	0.524	0.518	0.526	0.499	0.537	0.522	0.522

RO	AUC	0.566	0.568	0.565	0.597	0.581	0.569	0.586	0.558	0.576	0.586	0.575
	FMeasure	0.394	0.405	0.375	0.442	0.420	0.415	0.407	0.358	0.421	0.428	0.407
	GMean	0.523	0.530	0.505	0.564	0.544	0.539	0.537	0.490	0.547	0.555	0.533
	
	C. Avg.	0.494	0.501	0.482	0.534	0.515	0.508	0.510	0.469	0.515	0.523	0.505

Cluster	AUC	0.519	0.595	0.560	0.601	0.580	0.580	0.566	0.545	0.581	0.576	0.570
	FMeasure	0.306	0.343	0.358	0.357	0.332	0.270	0.301	0.368	0.387	0.339	0.445
	GMean	0.499	0.558	0.532	0.566	0.548	0.569	0.546	0.511	0.554	0.548	0.543
	
	C. Avg.	0.441	0.499	0.483	0.508	0.487	0.473	0.471	0.475	0.507	0.488	0.519

**Table 8:** Evaluation results of AMD-Neov dataset using different sampling strategies with three metrics across ten classification algorithms.

Method	Metric	Classifier										
		
		J48	3NN	NB	RF5	LOG	1NN	7NN	SMO	RF10	RBFNet	R. Avg.
PSO	AUC	0.681	0.659	0.661	0.662	0.678	0.656	0.694	0.628	0.686	0.672	0.668
	FMeasure	0.549	0.557	0.537	0.566	0.545	0.556	0.572	0.559	0.552	0.559	0.555
	GMean	0.622	0.628	0.619	0.643	0.626	0.630	0.648	0.637	0.631	0.631	0.632
	
	C. Avg.	0.617	0.615	0.605	0.624	0.616	0.614	0.638	0.608	0.623	0.621	0.618

RU	AUC	0.652	0.627	0.625	0.622	0.635	0.649	0.622	0.619	0.663	0.631	0.635
	FMeasure	0.549	0.526	0.524	0.534	0.519	0.531	0.543	0.529	0.561	0.539	0.536
	GMean	0.637	0.602	0.601	0.609	0.596	0.615	0.615	0.604	0.636	0.612	0.613
	
	C. Avg.	0.613	0.585	0.583	0.588	0.583	0.598	0.593	0.584	0.620	0.594	0.595

RO	AUC	0.643	0.643	0.646	0.659	0.635	0.655	0.638	0.632	0.660	0.657	0.647
	FMeasure	0.507	0.542	0.491	0.516	0.498	0.516	0.521	0.506	0.534	0.531	0.516
	GMean	0.602	0.629	0.589	0.610	0.599	0.612	0.612	0.598	0.624	0.623	0.610
	
	C. Avg.	0.584	0.605	0.575	0.595	0.577	0.594	0.590	0.579	0.606	0.603	0.591

Cluster	AUC	0.656	0.624	0.627	0.629	0.625	0.652	0.644	0.594	0.642	0.638	0.633
	FMeasure	0.551	0.524	0.502	0.538	0.506	0.521	0.546	0.504	0.536	0.537	0.527
	GMean	0.641	0.605	0.587	0.624	0.591	0.610	0.630	0.585	0.620	0.621	0.611
	
	C. Avg.	0.616	0.584	0.572	0.597	0.574	0.594	0.607	0.561	0.599	0.599	0.590

**Figure 4:**
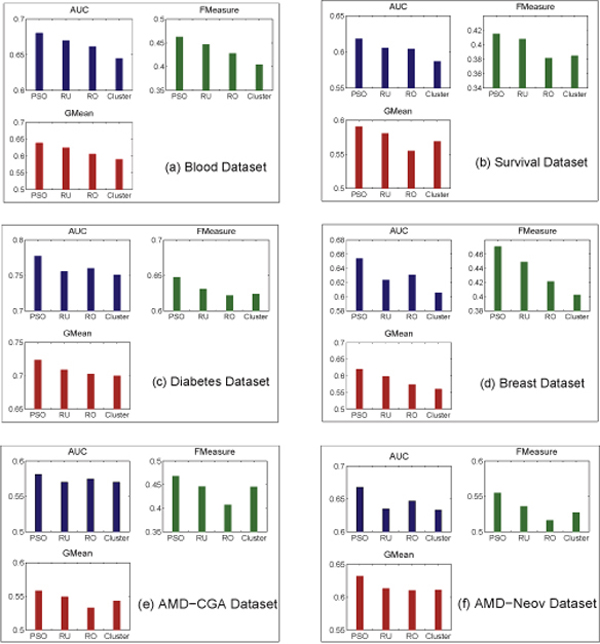
**Comparison of each sampling method with respect to different evaluation metrics**.

**Figure 5:**
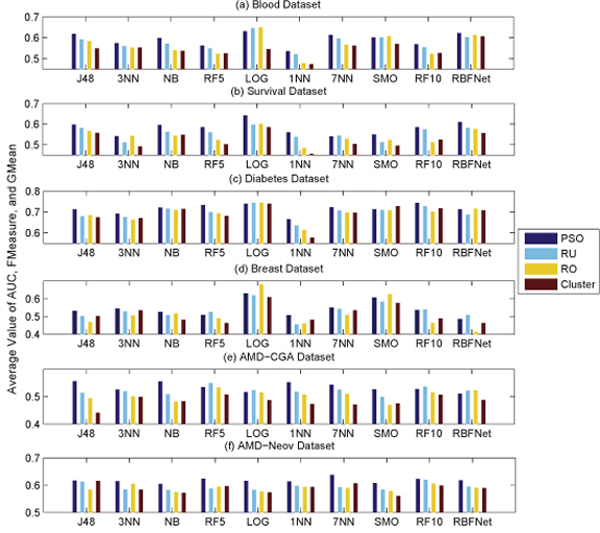
**Comparison of each sampling method with respect to different classification algorithms**.

By plotting the evaluation results with respect to different evaluation metrics (shown in Figure 4), we can see that the PSO hybrid achieved the highest accuracy within all six datasets. However, it is also clear that each evaluation metric gives a different evaluation indication. That is, a sampling method "A" performing worse than another method "B" according to certain evaluation metric may be superior to the method "B" using a different evaluation metric. By plotting the evaluation results with respect to different classification algorithms (in Figure 5), it is readily noticed that different classifiers also perform differently among these datasets. But within a given dataset, there seems to have certain data-classifier correlation regardless which type of the sampling method is used. Interestingly, logistic classifier seems to be quite effective, while 1NN appears to be the most unsuccessful one.

With above observation, it is clear that the evaluation of different data sampling strategies is compounded by different classification algorithms and evaluation metrics. Therefore, relying on a sole classifier or evaluation measure for imbalanced data sampling could potentially lead to the loss of generalization property. Caution should be drawn when a claim is made on the basis of a single type of classifier or evaluation metric.

## Conclusion

In this work, several popular sampling methods are investigated on imbalanced medical and biological data classification. A particle swarm based hybrid method is proposed to improve the overall classification accuracy. The experimental results on four medical datasets and a GWAS dataset illustrated the effectiveness of the proposed method. This is quantified in our experiments by using three evaluation metrics across 10 different classification algorithms.

The study demonstrates that with a proper modification feature selection algorithms can be tailored for imbalanced data sampling. In addition to being self-adaptable to different datasets, the proposed hybrid system is quite flexible, allowing different classifiers and evaluation components to be easily integrated for any specific problem at hand. The imbalanced data sampling problem is ubiquitous in clinical and medical diagnoses as well as gene function predication and protein classification [36,37]. The proposed hybrid system can not only recover the power of classifiers on imbalance data classification but also indicate the relative importance of samples from majority class in contrast to samples from minority class. This information could be used for further biological and medical investigations which may result in the discovery of new conditions or disease subtypes. We anticipate that such a hybrid formulation can provide a new means for tackling imbalanced data problems introduced in these applications.

## Availability

The PSO sampling system is implemented in Java and is available from: http://www.cs.usyd.edu.au/~yangpy/software/Sampling

## Competing interests

The authors declare that they have no competing interests.

## Authors' contributions

PY conceived the study and drafted the manuscript. PY and LX designed and implemented the algorithms, performed the experiments. BBZ, ZZ, and AYZ revised the manuscript critically and introduced the problem initially.

## Note

Other papers from the meeting have been published as part of *BMC Bioinformatics* Volume 10 Supplement 15, 2009: Eighth International Conference on Bioinformatics (InCoB2009): Bioinformatics, available online at http://www.biomedcentral.com/1471-2105/10?issue=S15.
